# Acting on Values: A Novel Intervention Enhancing Hedonic and Eudaimonic Well-Being

**DOI:** 10.1007/s10902-022-00585-4

**Published:** 2022-10-04

**Authors:** Agnieszka Bojanowska, Łukasz D. Kaczmarek, Beata Urbanska, Malwina Puchalska

**Affiliations:** 1 Department of Psychology, SWPS University of Social Sciences and Humanities, Chodakowska 19/31, 03-815 Warsaw, Poland; 2 Department of Psychology and Cognitive Science, Adam Mickiewicz University, Szamarzewskiego 89, 60-568 Poznan, Poland

**Keywords:** Well-being, Values, Satisfaction with life, Positive affect, Negative affect, Mindfulness, Interventions

## Abstract

Individuals increase well-being by acting on their values rather than merely endorsing them. We developed a novel intervention (“Acting on Values,” AoV), motivating individuals to initiate values-related behavior over four weeks. Building upon the theory of Basic Human Values, we expected that intervention recipients would increase their hedonic and eudaimonic well-being relative to a control group. We also expected the AoV interventions to cause similar effects as a mindfulness group. 783 volunteers (*N* = 268 completers) were assigned into three groups: AoV intervention, mindfulness, and a waiting list. Individuals who completed the AoV intervention achieved higher satisfaction with life, positive affect, and eudaimonic well-being, and lower negative affect than the control group. The well-being effects of the AoV intervention did not differ significantly from the mindfulness intervention effects. Our findings suggest that the AoV intervention is an efficacious method of increasing hedonic and eudaimonic well-being. It contributes to the diversity of well-being facilitation methods.

**Supplementary Information:**

The online version contains supplementary material available at 10.1007/s10902-022-00585-4.

## Introduction

Individuals tend to talk about their values more than they actually act on them (Sheldon & Krieger, 2014). This value importance/behavior gap is problematic because individuals become happier once they increase acting on their values (Sheldon & Krieger, 2014; Tessier et al., 2021). The value importance/behavior gap also suggests that individuals miss opportunities to enhance their lives via values engagement. Thus, most people might personally benefit from psychological assistance in the active pursuit of value-related goals.

Over the last two decades, an extensive literature has accumulated data regarding interventions that increase well-being or Positive Psychological Interventions (PPIs) (Seligman et al., 2005). However, none of these interventions has addressed the human values system explicitly. Most intervention studies have focused on increasing the hedonic aspect of well-being, e.g., subjective well-being (Diener, 2000). Many studies also examined eudaimonic well-being related to self-discovery and self-expression as reflected in engagement in personally relevant pursuits (Bolier et al., 2013; Weiss et al., 2016).

Building upon the theory of Basic Human Needs (Schwartz, 2012), we aimed to develop and examine the effects of a novel intervention that serves individuals in initiating more value-related actions in their daily life. We sought to account for a broad range of intervention outcomes, including subjective and eudaimonic well-being. Finally, we aimed to contribute to the literature by establishing how the novel intervention compares to a mindfulness-based intervention. This might provide more insight into whether behavioral engagement of values via an intervention (a more personalized approach) meets the effects of an intervention that has established efficacy but is less values-based.

Increasing the diversity of PPIs by engaging various positive emotions and positive behaviors is necessary for several reasons. Using diverse PPIs is likely to counter the effects of hedonic adaptation (Bao & Lyubomirsky, 2014). More extensive diversity in PPIs might also minimize several PPIs problems, such as differential effectiveness across cultures (Carr et al., 2020; Ng & Ong, 2021) or across personality traits and individual differences (Enko et al., 2021; Oltean et al., 2022; Wellenzohn et al., 2018). Finally, PPIs are more efficacious in multicomponent programs that engage several processes (van Agteren et al., 2021). In summary, the development and examination of this novel intervention might contribute to PPIs' repertoire expansion. It also helps refine and progress functional theories of values that address the value importance/behavior gap (Schwartz & Sortheix, 2018; Sheldon & Krieger, 2014).

## Basic Human Values

Values reflect what individuals consider important and worth pursuing in life (Schwartz & Sortheix, 2018; Schwartz et al., 2012). They are broad personal goals such as cultivating tradition, seeking stimulation and pleasure, or contributing to the welfare of others. There are individual differences in values, e.g., some values are essential to one person and unimportant to someone else. Values form a circular structure that reflects how they are related to each other: neighboring values share similar goals that can be realized through common behaviors, e.g., seeking achievements and power (Schwartz et al., 2012). Values on the opposite sides of the circle contradict one another, e.g., seeking stimulation vs. security. The circle of values forms higher-order groups. The most common division lists four higher-order constructs: openness to change (self-direction, stimulation, hedonism), self-enhancement (power, achievement), conservation (conformity, tradition), and self-transcendence (benevolence, universalism) (Schwartz et al., 2012).

Some values are considered healthy because they promote higher subjective well-being. Others hamper well-being and are considered unhealthy (Sortheix & Schwartz, 2018). Theorists suggest that healthy values reflect growth. In contrast, unhealthy values reflect deficiency and anxious self-protection. Openness to change and self-transcendence have proven to be the healthiest values (Bobowik et al., 2011; Cohen & Shamai, 2010; Sortheix & Schwartz, 2018). They motivate people to pursue new opportunities, satisfy their personal needs, and maintain good relationships with others. Self-enhancement and conservation were more often related to lower hedonic and eudaimonic indices of well-being (Bobowik et al., 2011; Bojanowska & Piotrowski, 2017 Sortheix & Schwartz, 2018). Yet, these effects were not consistent. Less frequent endorsement of unhealthy values is observed in numerous populations (Cieciuch, 2013). For instance, power is the least and benevolence the most endorsed value globally (Schwartz, 2007). Therefore, in our intervention, we left the participants the autonomy to act on any values they have (rather than suppressing some). We expected that healthy values would dominate their systems.

## Hedonic and Eudaimonic Well-Being

The possible effects of a values-based intervention cover a wide range of well-being outcomes. Accounting for the hedonic and eudaimonic perspectives is essential to capture the specific impacts of value-based interventions. Hedonic well-being is often represented by the theory of subjective well-being, which includes cognitive and affective components (Diener, 2000). The cognitive component refers to satisfaction with life, i.e., an individual's general belief that their life is similar to or different from their subjective ideal. The affective component emphasizes the abundance of positive emotions and the absence of negative emotions in daily life. Eudaimonic well-being represents the realization and expression of human and individual potential toward personal excellence (Kaczmarek, 2017; Waterman, 1990). This perspective focuses on developing a person's most essential skills and resources used to achieve self-concordant goals. Hedonists primarily strive toward specific feelings, whereas eudaimonists strive toward specific self-congruent goals (Kaczmarek, 2017). These two broad aspects of well-being are necessary to encompass the potentially unique effects of value-oriented interventions. Nonetheless, eudaimonic well-being is less studied as the PPIs' outcome relative to hedonic well-being (Koydemir et al., 2021). Developing new methods to increase eudaimonic well-being via interventions is particularly important because existing methods produce much smaller effects for eudaimonic well-being than subjective well-being (Koydemir et al., 2021).

## Acting on Values and its Effects on Well-Being

Theorists argue that individuals maximize the benefits of their value systems once they start to act upon them, i.e., "walking the talk" (Sheldon & Krieger, 2014). This has practical importance because research presents that a value importance/behavior gap exists in human values systems, such that individuals act on values ("walk") less than they explicitly endorse these values ("talk") (Sheldon & Krieger, 2014). A similar approach has been highlighted in the character strengths perspective (Seligman et al., 2005). Initiating actions that reflect personal moral traits leads to greater well-being (Schutte & Malouff, 2019).

Pursuing values is likely to increase well-being for several reasons. First, individuals are intrinsically rewarded for thoughts and actions congruent with their values and punished for incongruent thoughts and actions (Feather, 1996; Rohan, 2000; Schwartz & Sortheix, 2018). Thus, individuals acting on values more often in response to an intervention might experience more positive emotions (reward for congruence) and less negative emotions (less punishment for incongruence), making their lives more satisfying (Kim-Prieto et al., 2005). Second, individuals who act upon their values are more likely to experience the satisfaction of important goal achievement (Schwartz & Sortheix, 2018). This might result in increased eudaimonic well-being (Sheldon, 2002; Waterman et al., 2010). Moreover, individuals who initiate values-related behaviors in response to an intervention (e.g., helping others resulting from self-transcendence) are likely to generate more positive events in their lives. Thus, the AoV intervention recipients might derive more positive emotions (e.g., empathic joy) from the new events that would not take place otherwise.

These arguments support the development of PPIs based on individual value systems. The Theory of Basic Human Values (Schwartz, 1992), which focuses on personal beliefs and value-related actions, provides an excellent framework for developing tailored interventions. Such interventions would provide individuals with more opportunities to fill their attitude/behavior gap. This involves planning to act on their personal values and keeping to these plans (Hagger & Luszczynska, 2014). Therefore, our primary hypothesis was that an intervention that activates values would enhance subjective and eudaimonic well-being.

## Mindfulness Interventions

Mindfulness is a state of attention characterized by openness, acceptance, and an enhanced ability to respond to the present moment. It is the quality of awareness that arises through intentionally attending to the present moment experienced in a non-judgmental and accepting way (Kabat-Zinn, 1990). Mindfulness interventions enable individuals to experience the present moment with greater attention and awareness, fostering clear thinking, composure, compassion, and open-heartedness. Although mindfulness has its roots in Buddhist meditation, most evidence-based mindfulness interventions are now secular and practiced worldwide (Chen & Murphy, 2019). A standard mindfulness intervention is mindfulness-based stress reduction (MBSR; Kabat-Zinn, 1982), which includes a series of practices in increasing awareness of thoughts, breath, sounds, and other sensations. Mindfulness interventions range from 2 to 5-week brief mindfulness meditation interventions (Lim et al., 2015; Mrazek et al., 2013; Sass et al., 2019) to extensive 8-week programs (Vonderlin et al., 2020).

Meta-analyses have shown that mindfulness interventions increase satisfaction with life (Klussman et al., 2020), increase positive affect and decrease negative affect (Lindsay et al., 2018; Snippe et al., 2017), and enhance eudaimonic well-being (Bartlett et al., 2019; Vonderlin et al., 2020). Moreover, mindfulness interventions present the greatest efficacy in increasing well-being in clinical and non-clinical populations in comparison with distinct types of psychological interventions (van Agteren et al., 2021). Mindfulness interventions are also efficacious when delivered online (Howells et al., 2016; Kappen et al., 2019).

Therefore, mindfulness interventions are a good reference point for comparing the effectiveness of the value-related intervention. As stated in the literature, the effects of novel interventions should be compared with other well-established interventions (Heintzelman & Kushlev, 2020). Mindfulness interventions meet these criteria. They have been proven effective over various samples and contexts. A recent meta-analysis indicated that mindfulness interventions are the most efficacious interventions on a par with multicomponent PPIs delivered over an extended period (van Agteren et al., 2021). Additionally, mindfulness interventions are more universal than values-based interventions. Fewer individual preferences are considered in what individuals want to pursue and what actions to perform. Thus mindfulness interventions can be contrasted with a value-related intervention that is more personalized and has the potential for a better person-activity fit (Lyubomirsky & Layous, 2013).

## Online PPIs

There are several delivery vehicles for PPIs: face-to-face programs (e.g., Seligman et al., 2006), automated online methods, e.g., webpages (Seligman et al., 2005), or smartphone applications (Boucher et al., 2021; Rebedew, 2018). In online or app services, individuals interact with reading materials, pictures, or pre-recorded videos. The online format provides more standardization, easier intervention delivery, and progress tracking at the cost of communication quality and the absence of physical contact. PPIs with personal contact have been identified as producing stronger effects (Koydemir et al., 2021; Malouff & Schutte, 2017). However, more general work on interventions aimed at well-being (including PPIs) indicated that face-to-face and online interventions' formats produced similar results (van Agteren et al., 2021). This suggests that the findings regarding the intervention delivery methods are inconclusive. Moreover, previous research indicated that online PPIs are more effective when efforts are taken to engage individuals and, thus, prevent attrition (Parks, 2014).

## The Present Study

We aimed to examine the effects of a novel six-week-long online intervention to increase well-being via enhanced value-related actions in daily life. Building upon the Theory of Basic Values (Schwartz, 2012; Schwartz & Sortheix, 2018), and research documenting the benefits of acting on values (Sheldon & Krieger, 2014), we expected that stronger engagement in value-expressive behavior would increase hedonic and eudaimonic well-being. Furthermore, we aimed to test our intervention against two groups: a neutral control group and a mindfulness intervention group (Bartlett et al., 2019; Klussman et al., 2020; Snippe et al., 2017). This design allows examining whether the AoV intervention provides benefits relative to neutral conditions and other interventions. We addressed hedonic and eudaimonic well-being for outcomes, which is particularly important to capture the effects of values.

We primarily aimed to deliver the intervention face-to-face. However, the outbreak of the COVID-19 pandemic and the lockdown required that we turn to online methods. Thus, we tailored the intervention to be administered and completed online. Consequently, we tested this intervention using an intervention delivery vehicle that seems especially needed if similar crises and restrictions on face-to-face contact occur in the future.

Our approach based on values and well-being during a pandemic is essential if we consider an earlier observation that individuals modified their values during the lockdown and significantly declined all aspects of well-being (Bojanowska et al., 2021; Bonetto et al., 2021). Interventions that assist individuals in discovering new ways to act on their values despite restrictions might contribute to well-being preservation during pandemics. These interventions might also be worthwhile when the COVID-19 pandemic is over to strengthen individuals and further build their value-based resources (such as openness to change) that might buffer against other forms of social turmoil (Bojanowska et al., 2021).

## Method

### Participants

**Fig. 1:**
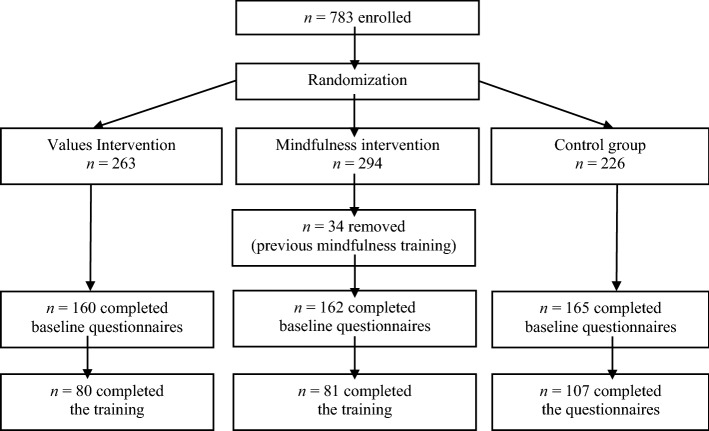
Flow diagram for study participants

A power analysis using G*Power 3.1 (Faul et al., 2009) indicated that detecting medium effect sizes of *f* = 0.25, with the power of 0.95, would require a final sample size of at least 251 participants for an ANCOVA with three groups and one covariate. To account for the expected dropout, we tripled the initial sample size. A total of 783 participants signed up for the experiment. They all gave informed consent to participate in the study and provided information regarding gender, age, education, and email address. Participants were randomly assigned to one of three groups: AoV, mindfulness, and the control group. Participants entered the mindfulness training group only if they had not received mindfulness training in the past. A total of 268 participants (34% of all enrolled and 55% of those who completed baseline questionnaires) completed the study (Fig. 1). Of the participants, 239 (89.2%) were female, and 29 (10.8%) were males. Their age was between 18 and 55 years (*M* = 34.09, *SD* = 9.49). The dropout size (Fig. 1) was comparable to other online studies (Melville et al., 2010; Morledge et al., 2013). The experiment was conducted between September 2020 and February 2021. Due to COVID-19 pandemic restrictions, we conducted the intervention online. We recruited participants among Polish psychology students and Facebook users (via Facebook ads). Psychology students received credits for participating in the study. We report how we determined our sample size, all data exclusions, all manipulations, and all measures in the study. Ethical Committee approved this study (SWPS University Ethics Committee, decision nr 38/2019).

### Procedure

#### Values Training

After completing the baseline questionnaires, respondents participating in values training received information regarding their four most important values (based on the PVQ questionnaire) along with a characteristic of these values. We used a framework that emphasizes ten values (Schwartz, 1992).

#### Mindfulness Training

Participants from the mindfulness training group received information about the level of their intentional attention measured with the Polish version of the Short Form Mindful Attention Awareness Scale (MAAS-SF-PL, Brown & Ryan, 2003; Radoń, 2014). There were four levels of interpretation: early beginner, beginner, intermediate, and advanced. The levels correspond to the points received in a questionnaire. Each level was described with behavioral language and indicated the pros and cons of each attention state and the advantages of participating in mindfulness training.

### Values Training Procedure

After getting feedback on their four most essential values, participants received the first reading material about the role of values according to Schwartz's theory. Their task was to plan how to act on their most important value over the upcoming week and put this plan into practice. Participants used an online journal to report what value they would be working on and what actions they planned to take (Supplement A). Participants received additional reading materials each week and planned to realize the subsequent values. Using their online diaries, they also reported how successfully they implemented their plans in the previous week and what actions they took.

We did not suggest the frequency of acting on values because participants planned activities that followed different patterns depending on the value. For instance, some values-related activities might be performed daily (e.g., being more helpful to others), weekly (going to the swimming pool), or less often (e.g., going to the opera). Moreover, some participants might perform different one-time tasks (e.g., buying geolocation trackers to increase children's outdoor security). Finally, some activities might be less plannable and more dependent on increased awareness and readiness to respond to emerging opportunities (deciding to actively participate in a social protest).

The materials that the respondents read each week addressed (1) the role of the values (according to Schwartz's theory); (2) the advantages of knowing one's value hierarchy and acting accordingly; (3) factors influencing which values are most important to people (including cultural factors); (4) the importance of regularity in acting on values. After the training, the participants completed post-test questionnaires. They also received feedback on how their well-being changed after the training.

### Mindfulness Intervention Procedure

Participants took a short form of a 4-week MBSR course, adjusted from an original 8-week program (Kabat-Zinn, 1990). After getting feedback on their mindfulness levels, participants received a 10-min guided meditation each week. They were encouraged to use daily situations to practice mindfulness and report them in an online journal. The meditations were: body scanning, sitting with the breath, sound, and walking meditation (Kabat-Zinn, 1990). The program started with mindfulness fundamentals (awareness of body sensation and breathing) and led towards more complex and challenging exercises (walking meditation).

As in the value intervention, participants completed the online diary reporting their engagement in the process (how often they planned to practice and how well they did in this task in the previous week). With each voice meditation, participants received one reading material per week (1) an introduction to mindfulness—what is mindfulness and how it is practiced; (2) advantages of practicing mindfulness—what changes participants can expect from regular practice; (3) mindfulness in different cultures—the Eastern and Western perspective on attentive awareness; (4) importance of the regular practice of mindfulness—information on the negative consequences of being in an unaware state of mind and how to enhance motivation to practice mindfulness. As in the value intervention, participants completed post-test questionnaires and received feedback.

The intervention plan is presented in Table 1.

**Table 1:** Intervention design

Week	Intervention	
	Acting on values	Mindfulness
	Baseline measurement	Baseline measurement
1	Feedback on personal values	Feedback on mindfulness levels
	Text material: The role of values	Text material: Introduction to mindfulness
	Applying the most important value in life	“Awareness of body sensation” meditation to practice during the next week
	Online diary	Online diary
2	Text material: Advantages of knowing the hierarchy of values	Text material: Advantages of practicing mindfulness
	Applying the second most important value in life	"Sitting with the breath" meditation to practice during the week
	Online diary	Online diary
3	Text material: Factors shaping the values (including culture)	Text material: Mindfulness in different cultures
	Applying the third most important value in life	Sound meditation to practice during the week
	Online diary	Online diary
4	Text material: The importance of the regular practice of acting upon values	Text material: Importance of regular practice of mindfulness
	Applying the fourth most important value in life	Walking meditation to practice during the week
	Online diary	Online diary
	Final measurement & feedback	Final measurement & feedback

### Intervention Engagement

Participants rated their intervention engagement on a scale from 1 ("very poor") to 10 ("very intense") after the study. We also monitored how many diaries the participants completed. The mean engagement was relatively high (*M* = 7.03, *SD* = 1.95), and 85% of the participants completed each assignment. We included all participants in the analysis regardless of their compliance. This is in line with the intention-to-treat rule ("once randomized, always analyzed"), providing an unbiased estimate of treatment effects (Gupta, 2011).

### Control Group Procedure

Participants assigned to the control group completed baseline questionnaires. After four weeks, they completed post-test questionnaires and received materials from the intervention they chose as a benefit.

### Measures

#### Eudaimonic Well-Being

We used the Questionnaire for Eudaimonic Well-being (Waterman et al., 2010; adapted into Polish by Kłym-Guba & Karaś (2018). It consists of 21 items (e.g., 'I believe I know what my strongest skills are and I try to develop them whenever possible'), with answers ranging from 1 ('strongly agree') to 7 ('strongly disagree'). Higher scores indicated higher eudaimonic well-being. This scale had satisfactory reliability with Cronbach's *α* = 0.84 and McDonald's *ω* = 0.84.

#### Positive and Negative Affect

Positive affect and negative affect were measured using the Positive and Negative Affect Schedule (Watson et al., 1988; Bojanowska & Zalewska, 2015). This measure comprises a list of ten adjectives referring to positive (e.g., interested, excited) and ten adjectives referring to negative (e.g., guilty, ashamed) affective states experienced over the previous two weeks. Participants responded on a scale from 1 ('slightly or not at all') to 5 ('extremely'). Higher scores represented higher intensity of affect. The scales had satisfactory reliability with Cronbach's *α* = 0.70 and McDonald's *ω* = 0.77 for positive affect and Cronbach's *α* = 0.89 and McDonald's *ω* = 0.89 for negative affect.

*Satisfaction with Life.* We used the Satisfaction with Life Scale (SWLS) (Diener et al., 1985; Bojanowska & Piotrowski, 2017) to assess participants' global evaluation of their life ('In most ways, my life is close to my ideal') on a scale from 1 ('I definitely disagree') to 7 ('I definitely agree'). Higher scores represent higher satisfaction with life. This scale had satisfactory reliability with Cronbach's α = 0.83 and McDonald's *ω* = 0.84.

### Analytical strategy

We used a 3 (group: the AoV intervention, the mindfulness intervention, and control) × 2 (gender) ANCOVA with post-test values as the outcome while controlling for baseline values as a covariate. Such models assess the difference in post-test means while accounting for pre-test values, which provides more statistical power (Clifton & Clifton, 2019; Van Breukelen, 2006). We present partial eta-squared (η^2^) with η^2^ > 0.14 for large, η^2^ > 0.07 for medium, and η^2^ > 0.02 for small effect sizes. We included in the supplementary materials a repeated-measures ANOVA, which does not adjust the outcomes for pre-test values (Supplement B). We performed these analyses with SPSS 26.0 (IBM, USA).

Furthermore, we tested the intervention's effects equivalence (Lakens, 2017). Accounting for equivalence allows testing if the effects are robust enough to differ from 0 to a meaningful extent, i.e., beyond a predefined range of equivalence. Thus, the equivalence test goes beyond the traditional testing if the difference is at least higher than 0. This procedure is identical to testing if the effect's 90% two-sided confidence interval falls entirely within the set equivalence range. The equivalence lower and upper bounds represent effects considered too small to be meaningful. We set the Cohen's *d* equivalence bounds of Δ_*L*_ =  − 0.23 and Δ_*U*_ = 0.23. Such positive interventions' overall effect size was identified in a meta-analysis that accounted for mindfulness interventions (Koydemir et al., 2020)*.* Consequently, we tested if each increase in outcomes from the baseline to the post-test was meaningful relative to the effects observed in other studies. We tested equivalence using jamovi software (The jamovi project, 2021) and the TOSTER module (Lakens, 2017).

## Results

*Preliminary analyses.* Groups did not differ in baseline satisfaction with life, *F* (2, 265) = 1.92, *p* = 0.15, η^2^ = 0.01, eudaimonic well-being, *F* (2, 265) = 0.16, *p* = 0.85, η^2^ < 0.01, and negative affect, *F* (2, 265) = 0.80, *p* = 0.45, η^2^ = 0.01. There were small baseline differences in positive affect, *F* (2, 265) = 3.58, *p* = 0.03, η^2^ = 0.03. Thus, we accounted for baseline in determining intervention outcomes.

The frequency of values endorsement is presented in Table 2. We present raw and ipsative scores of values. Ipsatization is a correction of variables by their common component, which involves within-individual centering. As recommended (Schwartz, 1992), we calculated the ipsative scores by subtracting an overall individual mean of all value items from every specific value index based on subsets of these items. We present raw and ipsative scores because simulation studies indicate that raw and ipsatized scores have their advantages and limitations; thus, both are best presented (Rudnev, 2021). Participants tended to endorse openness to change and oelf-transcendence values more often (higher raw score means and ipsative scores are above 0). They endorsed self-enhancement and conservation less often (lower raw score means and ipsative scores below 0).

**Table 2:** Values endorsement. Mean and standard deviations (in brackets) for each higher-order value

	Openness to change	Self-enhancement	Conservation	Self-transcendence
Raw scores	4.43(.71)	3.45(.85)	3.79(.72)	4.93(.62)
Ipsative scores	.27(.61)	− .70(.67)	− .38(.47)	.77(.42)

Higher scores reflect stronger values endorsement

**Fig. 2:**
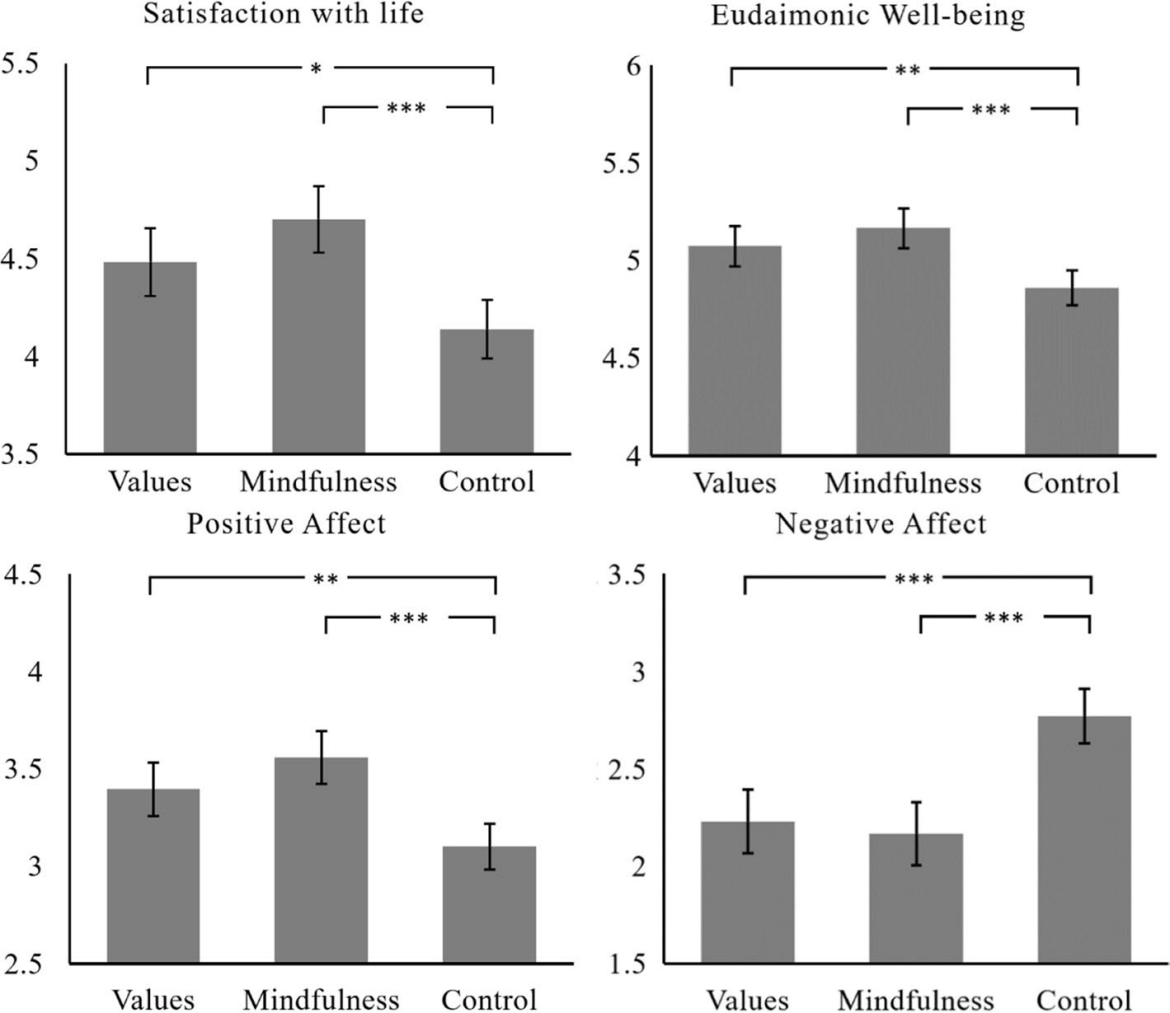
The effects of the interventions on well-being. *Note* Covariate-adjusted means. Post hoc comparison with Bonferroni correction. Error bars represent 95% CIs. *p < .05, **p < .01, ***p < .001

*Intervention effects.* We found that individuals involved in the AoV intervention and those involved in the mindfulness intervention achieved higher levels of satisfaction with life, *F* (2, 264) = 12.27; *p* < 0.001; η^2.^ = 0.09), positive emotions, *F* (2, 264) = 13.26, *p* < 0.001, η^2.^ = 0.09), and eudaimonic well-being, *F* (2, 264) = 10.66, *p* < 0.001, η^2.^ = 0.08, and lower levels of negative emotions, *F* (2, 264) = 19.39, *p* < 0.001; η^2.^ = 0.13, than the control group (Fig. 2). These effects were moderate in size. There were no significant differences between the effects of the AoV and the mindfulness intervention.

The equivalence analysis indicated similar results (Table 3). There were significant differences in pre- vs. post-test outcomes. As expected, the levels from pre-test to post-test were generally significantly different and non-equivalent in the interventions groups. Repeated-measures ANOVA that did not adjust for the baseline produced similar results (Supplement B).

**Table 3:** Descriptive statistics and equivalence tests

Group	Variable	*M* (*SD*)		Paired *t*-test	Equivalence *t*-test (TOST)		Statistical interpretation	
		Pretest	Posttest		Lower bound	Upper bound	Difference	Equivalence
Acting on values	Life satisfaction	4.15(1.06)	4.63(1.22)	5.33^***^	7.39^**^	3.28	Yes	No
	Eudaimonic WB	4.86(0.74)	5.06(0.80)	3.10^***^	5.16^***^	1.04	Yes	No
	Positive emotions	3.34(0.71)	3.49(0.66)	1.81^a^	3.87^***^	− .24	Marginally	No
	Negative emotions	2.67(0.91)	2.21(0.86)	− 4.66^***^	− 2.60	− 6.71^***^	Yes	No
Mindfulness	Life satisfaction	4.00(1.21)	4.72(1.21)	6.22^***^	8.29^***^	4.15	Yes	No
	Eudaimonic WB	4.91(0.76)	5.2(0.76)	5.96^***^	8.03^***^	3.89	yes	No
	Positive emotions	3.10 (0.75)	3.53(0.73)	6.22^***^	8.29^***^	4.15	Yes	No
	Negative emotions	2.63(0.81)	2.12(0.90)	− 6.84^***^	− 4.77	− 8.91^***^	Yes	No
Control	Life satisfaction	3.82(1.21)	4.02(1.18)	2.27^*^	4.65^***^	− 0.11	Yes	No
	Eudaimonic WB	4.85(0.79)	4.84(0.80)	− 0.23	2.14^*^	− 2.61^**^	No	Yes
	Positive emotions	3.07(0.73)	3.05(0.78)	− 0.18	2.19^*^	− 2.56^**^	No	Yes
	Negative emotions	2.79(0.95)	2.82(0.98)	.39	− 1.99^*^	2.76^**^	No	Yes

*df*_AoV_ = 79, *df*_mindfulness_ = 80, *df*_cont_ = 106

^a^*p* < .07, *p < .05, **p < .01, ***p < .001

## Discussion

We developed and examined a novel values-based intervention aimed at increasing well-being. As expected, individuals who completed the AoV intervention achieved higher subjective and eudaimonic well-being than a neutral control group. Namely, the AoV intervention recipients were more satisfied with their life, enjoyed more self-expression (eudaimonic well-being), and felt more positive and less negative affect than individuals who did not initiate any intervention. Moreover, the results of the AoV intervention were comparable to the effects of well-established mindfulness intervention. Our findings also indicated the feasibility of the novel AoV intervention relative to the mindfulness intervention, as their dropout rates were similar. We tested this intervention under restrictions on daily activities during the COVID-19 lockdown.

We provided systematic and well-controlled evidence that individuals who intensify acting on their values are more likely to increase their well-being. This finding contributes to the Basic Human Values framework discussion regarding the relationship between values and well-being (Sagiv & Schwartz, 2000). Our results support the existence of the value importance/behavior gap (Sheldon & Kireger, 2014) because participants realized their well-being potential after receiving more motivation and assistance in acting upon their values, i.e., walking the talk. This change indicates the significance of the gap as well as the feasibility and relative ease of reducing it by interventions. Finally, our findings also addressed classical philosophical (Kaczmarek, 2017) and psychological (e.g., Waterman et al., 2010) accounts of eudaimonia that emphasize that individuals pursue happiness effectively when they pursue what they consider essential in life.

We found that the novel AoV intervention produced similar outcomes as a well-established mindfulness intervention. Both interventions had comparable moderate effects on each well-being facet. It indicates that the AoV intervention meets the domain standards. This finding also corroborates previous meta-analyses indicating that mindfulness interventions produce results comparable to PPIs delivered over an extended period (van Agteren et al., 2021). Having intervention alternatives is essential as the hedonic adaptation theories emphasize the need for different activities and experiences in building enduring well-being (Bao & Lyubomirsky, 2014; Wilson & Gilbert, 2008). Our effort also follows recommendations to increase new PPI development's scientific quality (Heintzelman & Kushlev, 2020). We present that further work on the effects of PPIs might benefit from comparing interventions against each other.

We covered a broad range of well-being facets from affect to cognition and from the hedonic to eudaimonic perspective. However, we observed similar effects of the interventions for each well-being measure. This reveals the strength of the AoV intervention because the effects of PPIs are usually less pronounced for eudaimonic well-being than for subjective well-being (Koydemir et al., 2021). Moreover, these uniform outcomes across each well-being facet address whether distinguishing between different aspects of well-being makes practical sense (e.g., Disabato et al., 2016). We found that interventions operated in the same direction and had similar strength in their effects on each well-being facet. This supports the notion that they might operate together in daily life. Of note, we observed the strongest effect of the intervention (on the verge of moderate and strong effects) for negative affect. It might suggest that the AoV intervention might be the most effective in reducing distress. This finding might address new questions regarding the primary role of emotions in the PPI' working mechanisms (Moskowitz et al., 2021).

We focused on the general positive effect of walking the talk identified in previous studies (Sheldon & Krieger, 2014; Tessier et al., 2021). Thus, participants were free to act upon any of their dominant values. Most of our participants more frequently endorsed openness to change and self-transcendence. These two groups of values are considered healthier because they reflect a growth orientation, whereas self-enhancement and conservation (less frequently endorsed in our sample) might be driven more by managing anxiety and self-protection (Sortheix & Schwartz, 2018). Such a structure of value endorsement, especially the less frequent endorsement of self-enhancement, is observed in numerous populations (Cieciuch, 2013) and can be deemed representative of the broader population.

Our study also provides a new replication of the benefits of mindfulness interventions (Bartlett et al., 2019; Klussman et al., 2020; Lindsay et al., 2019; Snippe et al., 2017; Vonderlin et al., 2020). We used this intervention primarily as a benchmark for our novel intervention. However, the results contribute to mindfulness research. First, we replicated previous findings that practicing mindfulness provides benefits for well-being. Notably, whereas most previous studies used more extended mindfulness programs, we present moderate benefits reached in a four-week mindfulness training. Moreover, we report evidence that mindfulness interventions increase eudaimonic well-being, a facet of well-being studied as the outcome of mindfulness interventions less often. Finally, few studies compared mindfulness training with other interventions. Thus, we present that the mindfulness intervention does not offer superior effects when used to increase well-being. The effects were moderate and comparable to another intervention that did not focus on mindfulness.

### Practical Implications

This study has some practical implications. First, we offer a novel intervention that practitioners of psychology might use with clients to improve their well-being. As diversity is essential for PPIs to work effectively (Bao & Lyubomirksy, 2014), the AoV intervention might be a worthwhile addition to the PPIs repertoire. Second, despite the AoV intervention devised as a life-enhancing method with a positive focus, we observed its particular efficacy in reducing negative feelings. This might indicate that the AoV intervention might be used to counter negative feelings in non-clinical samples. Third, we present evidence that motivating individuals toward a life more infused with values does not provide costs to their well-being. In contrast, individuals activating their values enjoyed these activities and were more satisfied with life. Practitioners can use this finding to build positive attitudes and expectations towards values and value-related actions among their clients. This might be considered from the sociotherapeutic perspective that often emphasizes the development of values systems. Fourth, our findings regarding values and mindfulness might be relevant to Acceptance and Commitment Therapy because value-expressive behaviors and mindfulness training constitute this approach's base (Hayes et al., 2006). Finally, our work provides some of the earliest interventions whose effectiveness was tested in the context of the COVID-19 pandemic (Dennis et al., 2021; Grasedieck, 2021; Xiaomei et al., 2020). Thus, we recommend the AoV intervention (as the mindfulness intervention) to practitioners who need evidence for an intervention to work in this particular social context, e.g., using a format that respects social distancing or an intervention that individuals perform despite pandemic restrictions on daily behavior.

### Limitations and Future Directions

This study has several limitations. First, we conducted this study in Poland. Due to national differences in dominating values and their link with well-being, the outcomes might differ in other countries (Sagiv & Schwartz, 2000). Second, we delivered interventions online. Further studies might test how the technology moderates the outcomes of this intervention. It is not unlikely that the effects might be more pronounced using face-to-face sessions because previous analyses indicated that personal contact increases the influence of PPIs (Koydemir et al., 2021; Malouff & Schutte, 2017). Third, most of the volunteers who responded to our invitation were female. With few men in our sample, we could not reliably test whether gender moderated the effects of the intervention. Further studies might examine whether these findings generalize to men. Fourth, we provided the intervention during the COVID-19 lockdown. This pandemic had substantial adverse effects on well-being and transformed some personal values, e.g., by reducing hedonistic pursuits (Bojanowska et al., 2021). We know little whether the effects of the AoV intervention would be the same if individuals had higher baseline levels of well-being and if extraordinary circumstances did not influence participants' values. Fifth, despite the random assignment to groups, we observed a minor baseline difference in positive affect. Our analytic approach aimed to minimize these effects by adjusting the outcomes for what might be expected if both groups had equal starting levels of positive affect (Clifton & Clifton, 2019; Van Breukelen, 2006). Nevertheless, this analytical method does not rule out the possibility that it might have been more difficult for individuals in the AoV group to improve on positive affect if the participants in the other groups had more room to improve (Pearl, 2016). Sixth, we experienced a considerable dropout from the intervention. Although the dropout rate was comparable to other intervention studies, some methods to reduce dropout might be employed. For instance, our intervention might be more tailored to smartphone use as smartphones become increasingly popular in delivering PPIs (e.g., Howells et al., 2016). Seventh, we tested this intervention in a non-clinical sample. Thus, this efficacy test does not generalize to clinical samples, e.g., depressed or anxious individuals. Mindfulness interventions and many PPIs have been efficacious in clinical settings (Geerling et al., 2020; Lai et al., 2019). Thus, further studies might seek ways to adjust this intervention to facilitate well-being among individuals representing clinical groups. Eight, the control group did not perform any activity. They may have experienced lower well-being, especially due to the COVID-19 lockdown and restrictions. Further testing of the AoV intervention might benefit from a comparison with a group performing a neutral or a placebo activity. Ninth, mindfulness was a fixed schedule intervention, whereas the AoV intervention allowed for a broader range of behavioral patterns. Further studies might dissect and optimize this behavioral aspect by manipulating the frequency of AoV and mindfulness interventions. Tenth, we used a mindfulness intervention as a reference point. However, it would also be worth comparing the AoV intervention's effects with other interventions, including those that provide less pronounced effects. This would allow observing more relative benefits of the AoV intervention and presenting our intervention within a broader context of existing research and practice. Finally, further studies might use designs powered enough to address the engagement of risky values. Acting upon some values might be less or inversely related to well-being, e.g., power-seeking (Sarkova et al., 2013). Such studies might identify if the AoV intervention is likely to backfire for individuals endorsing specific values despite its general positive effect on a group level. This might add to the literature regarding individual differences in predicting PPIs outcomes (e.g., Enko et al., 2021; Oltean et al., 2022; Wellenzohn et al., 2018).

## Conclusions

We presented a successful development of a new method to increase well-being, i.e., the AoV intervention. This method increases the diversity of PPIs and aims to engage individuals in personally valued activities. Such methods are crucial in everyday life for individuals who cultivate their well-being, including those under the burden of a pandemic lockdown. Our work contributes to the development of the PPIs as a validated instrument of personal change.

## Supplementary Information

Below is the link to the electronic supplementary material.

Supplementary file1 (DOCX 13 kb)

Supplementary file2 (DOCX 38 kb)

## Data Availability

Study’s data available at: https://osf.io/e9xr7/

## References

[CR2] Bao KJ, Lyubomirsky S, Parks AC, Schuller SM (2014). Making happiness last: Using the hedonic adaptation prevention model to extend the success of positive interventions. The Wiley-Blackwell handbook of positive psychological interventions.

[CR3] Bartlett L, Martin A, Neil AL, Memish K, Otahal P, Kilpatrick M, Sanderson K (2019). A systematic review and meta-analysis of workplace mindfulness training randomized controlled trials. Journal of Occupational Health Psychology.

[CR81] Bobowik, M., Basabe, N., Paez, D., Jimenez, A., & Bilbao, M. A. (2011). Personal values and well-being among Europeans, Spanish natives, and immigrants to Spain: Does the culture matter? *Journal of Happiness Studies,**12,* 401–419. 10.1007/s10902-010-9202-1

[CR80] Bolier, L., Haverman, M., Westerhof, G. J., Riper, H., Smit, F., & Bohlmeijer, E. (2013). Positive psychology interventions: a meta-analysis of randomized controlled studies. *BMC Public Health,**13,* 119. 10.1186/1471-2458-13-119 10.1186/1471-2458-13-119 PMC3599475 23390882

[CR4] Bojanowska, A., Kaczmarek, Ł. D., Kościelniak, M., & Urbańska, B. (2021). Changes in values and well-being amidst the COVID-19 pandemic in Poland. *PLOS One,**16*(9): e0255491. 10.1371/journal.pone.0255491 10.1371/journal.pone.0255491 PMC8443041 34525095

[CR5] Bojanowska A, Piotrowski K (2017). Values and psychological well-being among adolescents—are some values 'healthier' than others?. European Journal of Developmental Psychology.

[CR6] Bonetto E, Dezecache G, Nugier A, Inigo M, Mathias JD, Huet S, Dambrun M (2021). Basic human values during the COVID-19 outbreak, perceived threat and their relationships with compliance with movement restrictions and social distancing. PLoS ONE.

[CR7] Boucher EM, McNaughton EC, Harake N, Stafford JL, Parks AC (2021). The impact of a digital intervention (Happify) on loneliness during COVID-19: Qualitative focus group. JMIR Mental Health.

[CR8] Brown KW, Ryan RM (2003). The benefits of being present. Mindfulness and its role in well-being. Journal of Personality and Social Psychology.

[CR9] Carr, A., Cullen, K., Keeney, C., Canning, C., Mooney, O., Chinseallaigh, E., & O'Dowd, A. (2020). Effectiveness of positive psychology interventions: a systematic review and meta-analysis. *The Journal of Positive Psychology*, 1–21.

[CR10] Chen S, Murphy D (2019). The mediating role of authenticity on mindfulness and well-being: A cross-cultural analysis. Asia Pacific Journal of Counselling and Psychotherapy.

[CR11] Cieciuch J (2013). Kształtowanie się systemu wartości od dzieciństwa do wczesnej dorosłości [The formation of a value system from childhood to early adulthood.].

[CR12] Clifton L, Clifton DA (2019). The correlation between baseline score and post-intervention score, and its implications for statistical analysis. Trials.

[CR82] Cohen, A., & Shamai, O. (2010). The relationship between individual values, psychological well-being, and organizational commitment among Israeli police officers. *International Journal of Police Strategies & Management*, *33*, 30–51. 10.1108/13639511011020584

[CR15] Dennis A, Ogden J, Hepper EG (2021). Evaluating the impact of a time orientation intervention on well-being during the COVID-19 lockdown: Past, present or future?. The Journal of Positive Psychology.

[CR16] Diener E (2000). Subjective well-being: The science of happiness and a proposal for a national index. American Psychologist.

[CR17] Disabato DJ, Goodman FR, Kashdan TB, Short JL, Jarden A (2016). Different types of well-being? A cross-cultural examination of hedonic and Eudaimonic well-being. Psychological Assessment.

[CR18] Enko, J., Behnke, M., Dziekan, M., Kosakowski, M., & Kaczmarek, L. D. (2021). Gratitude texting touches the heart: Challenge/threat cardiovascular responses to gratitude expression predict self-initiation of gratitude interventions in daily Life. *Journal of Happiness Studies*, *22*, 49–69.

[CR19] Faul F, Erdfelder E, Buchner A, Lang AG (2009). Statistical power analyses using G* Power 3.1: Tests for correlation and regression analyses. Behavior Research Methods.

[CR20] Feather NT, Seligman C, Olson JM, Zanna MP (1996). Values, deservingness, and attitudes toward high achievers: Research on tall poppies. The Ontario symposium: The psychology of values.

[CR21] Grasedieck, J. (2021). *Effectiveness of a gratitude app on happiness and distress of employees during the COVID-19 pandemic* (Bachelor's thesis, University of Twente).

[CR22] Gupta SK (2011). Intention-to-treat concept: A review. Perspectives in Clinical Research.

[CR23] Hagger MS, Luszczynska A (2014). Implementation intention and action planning interventions in health contexts: State of the research and proposals for the way forward. Applied Psychology: Health and Well-Being.

[CR24] Hayes SC, Luoma JB, Bond FW, Masuda A, Lillis J (2006). Acceptance and commitment therapy: Model, processes and outcomes. Behaviour Research and Therapy.

[CR25] Heintzelman SJ, Kushlev K (2020). Emphasizing scientific rigor in the development, testing, and implementation of positive psychological interventions. The Journal of Positive Psychology.

[CR27] Howells A, Ivtzan I, Eiroa-Orosa FJ (2016). Putting the 'app'in happiness: A randomised controlled trial of a smartphone-based mindfulness intervention to enhance well-being. Journal of Happiness Studies.

[CR29] Kabat-Zinn J (1990). Full catastrophe living: Using the wisdom of your body and mind to face stress, pain, and illness.

[CR85] Kabat-Zinn, J. (1982). An outpatient program in behavioral medicine for chronic pain patients based on the practice of mindfulness meditation: Theoretical considerations and preliminary results. *General Hospital Psychiatry,**4*(1), 33-47. 10.1016/0163-8343(82)90026-3 7042457

[CR30] Kaczmarek LD, Zeigler-Hill V, Shackelford TK (2017). Eudaimonic motivation. Encyclopedia of personality and individual differences.

[CR31] Kappen G, Karremans JC, Burk WJ (2019). Effects of a short online mindfulness intervention on relationship satisfaction and partner acceptance: The moderating role of trait mindfulness. Mindfulness.

[CR32] Kim-Prieto C, Diener E, Tamir M, Scollon C, Diener M (2005). Integrating the diverse definitions of happiness: A time-sequential framework of subjective well-being. Journal of Happiness Studies.

[CR33] Klussman K, Curtin N, Langer J, Nichols AL (2020). Examining the effect of mindfulness on well-being: Self-connection as a mediator. Journal of Pacific Rim Psychology.

[CR34] Kłym-Guba, M., & Karaś, D. (2018). Polish version of the Questionnaire for Eudaimonic Well-Being – three factors rather than one. *Health Psychology Report,**6,*273–283.

[CR35] Koydemir S, Sökmez AB, Schütz A (2021). A meta-analysis of the effectiveness of randomized controlled positive psychological interventions on subjective and psychological well-being. Applied Research in Quality of Life.

[CR36] Lai ST, Lim KS, Low WY, Tang V (2019). Positive psychological interventions for neurological disorders: A systematic review. The Clinical Neuropsychologist.

[CR87] Lakens, D. (2017). Equivalence tests: A practical primer for t tests, correlations, and meta-analyses. *Social Psychological and Personality Science,**8*(4), 355-362. 10.1177/1948550617697177 PMC5502906 28736600

[CR38] Lim D, Condon P, DeSteno D (2015). Mindfulness and compassion: An examination of mechanism and scalability. PLoS ONE.

[CR39] Lindsay EK, Chin B, Greco CM, Young S, Brown KW, Wright A, Smyth JM, Burkett D, Creswell JD (2018). How mindfulness training promotes positive emotions: Dismantling acceptance skills training 7in two randomized controlled trials. Journal of Personality and Social Psychology.

[CR40] Lyubomirsky S, Layous K (2013). How do simple positive activities increase well-being?. Current Directions in Psychological Science.

[CR41] Malouff JM, Schutte NS (2017). Can psychological interventions increase optimism? A meta-analysis. The Journal of Positive Psychology.

[CR43] Melville KM, Casey LM, Kavanagh DJ (2010). Dropout from Internet-based treatment for psychological disorders. The British Journal of Clinical Psychology.

[CR44] Morledge TJ, Allexandre D, Fox E, Fu AZ, Higashi MK, Kruzikas DT, Pham SV, Reese PR (2013). Feasibility of an online mindfulness program for stress management—A randomized, controlled trial. Annals of Behavioral Medicine: A Publication of the Society of Behavioral Medicine.

[CR45] Moskowitz JT, Cheung EO, Freedman M, Fernando C, Zhang MW, Huffman JC, Addington EL (2021). Measuring positive emotion outcomes in positive psychology interventions: A literature review. Emotion Review.

[CR46] Mrazek MD, Franklin MS, Phillips DT, Baird B, Schooler JW (2013). Mindfulness training improves working memory capacity and GRE performance while reducing mind wandering. Psychological Science.

[CR47] Ng, W., & Ong, K. R. (2021). Using positive psychological interventions to improve well-being: Are they effective across cultures, for clinical and non-clinical samples?. *Journal of Contemporary Psychotherapy*, 1–9.

[CR48] Oltean, L. E., Miu, A. C., Șoflău, R., & Szentágotai-Tătar, A. (2022). Tailoring gratitude interventions. How and for whom do they work? The potential mediating role of reward processing and the moderating role of childhood adversity and trait gratitude. *Journal of Happiness Studies*, 1–24.

[CR49] Parks AC (2014). A case for the advancement of the design and study of online positive psychological interventions. The Journal of Positive Psychology.

[CR50] Pearl, J. (2016). Lord's paradox revisited–(oh Lord! Kumbaya!). *Journal of Causal Inference*, *4*(2). Również pojęcie „pomyślność” (*blessing*) pochodzi z często badanej pozytywnej interwencji dostrz

[CR51] Radoń S (2014). Pięciowymiarowy kwestionariusz uważności: Polska adaptacja [The five-dimensional mindfulness questionnaire: a Polish adaptation.]. Roczniki Psychologiczne.

[CR52] Rebedew D (2018). Five mobile apps for mindfulness. Family Practice Management.

[CR53] Rohan MJ (2000). A rose by any name? The values construct. Personality and Social Psychology Review.

[CR54] Rudnev M (2021). Caveats of non-ipsatization of basic values: A review of issues and a simulation study. Journal of Research in Personality.

[CR55] Sass SM, Early LM, Long L, Burke A, Gwinn D, Miller P (2019). A brief mindfulness intervention reduces depression, increases nonjudgment, and speeds processing of emotional and neutral stimuli. Mental Health & Prevention.

[CR57] Schwartz SH, Zanna M (1992). Universals in the content and structure of values: Theoretical advances and empirical tests in 20 countries. Advances in experimental social psychology.

[CR58] Schwartz SH (2007). Value orientations: Measurement, antecedents and consequences across nations. Measuring Attitudes Cross-Nationally: Lessons from the European Social Survey.

[CR59] Schwartz SH (2012). An overview of the Schwartz theory of basic values. Online Readings in Psychology and Culture.

[CR60] Schwartz SH, Cieciuch J, Vecchione M, Davidov E, Fischer R, Beierlein C, Ramos A, Verkasalo M, Lönnqvist J-E, Demirutku K, Dirilen-Gumus O, Konty M (2012). Refining the theory of basic individual values. Journal of Personality and Social Psychology.

[CR61] Schwartz SH, Sortheix FM, Diener E, Oishi S, Tay L (2018). Values and subjective well-being. Handbook of well-being.

[CR62] Seligman ME, Rashid T, Parks AC (2006). Positive Psychotherapy. American Psychologist.

[CR86] Schutte, N. S., & Malouff, J. M. (2019). The impact of signature character strengths interventions: A meta-analysis. *Journal of Happiness Studies,**20*(4), 1179-1196.

[CR63] Seligman ME, Steen TA, Park N, Peterson C (2005). Positive psychology progress: Empirical validation of interventions. American Psychologist.

[CR64] Sheldon KM, Deci EL, Ryan RM (2002). The self-concordance model of healthy goal striving: When personal goals correctly represent the person. Handbook of self-determination research.

[CR65] Sheldon KM, Krieger LS (2014). Walking the talk: Value importance, value enactment, and well-being. Motivation & Emotion.

[CR67] Snippe E, Dziak JJ, Lanza ST (2017). The shape of change in perceived stress, negative affect, and stress sensitivity during mindfulness-based stress reduction. Mindfulness.

[CR68] Tessier J, Joussemet M, Kurdi V (2021). Adolescents "walking the talk": How value importance and enactment relate to well-being and risk-taking. Motivation & Emotion.

[CR69] The jamovi project (2021). jamovi (Version 1.6) [Computer Software]. Retrieved from https://www.jamovi.org

[CR70] van Agteren J, Iasiello M, Lo L, Bartholomaeus J, Kopsaftis Z, Carey M, Kyrios M (2021). A systematic review and meta-analysis of psychological interventions to improve mental well-being. Nature Human Behaviour.

[CR71] Van Breukelen GJ (2006). ANCOVA versus change from baseline had more power in randomized studies and more bias in nonrandomized studies. Journal of Clinical Epidemiology.

[CR72] Vonderlin R, Biermann M, Bohus M (2020). Mindfulness-based programs in the workplace: A meta-analysis of randomized controlled trials. Mindfulness.

[CR73] Waterman AS, Schwartz SJ, Zamboanga BL, Ravert RD, Williams MK, Bede Agocha V, Brent Donnellan M (2010). The Questionnaire for Eudaimonic Well-Being: Psychometric properties, demographic comparisons, and evidence of validity. Journal of Positive Psychology.

[CR83] Waterman, A. S. (1990). Personal expressiveness: Philosophical and psychological foundations. *Journal of Mind and Behavior,**11,* 47–74.

[CR74] Watson D, Clark LA, Tellegen A (1988). Development and validation of brief measures of positive and negative affect: The PANAS scales. Journal of Personality and Social Psychology.

[CR88] Weiss, L.A., Westerhof, G.J., Bohlmeijer, E.T. (2016) Can We Increase Psychological Well-Being? The Effects of Interventions on Psychological Well-Being: A Meta-Analysis of Randomized Controlled Trials. *PLOS One,**11*(6): e0158092. 10.1371/journal.pone.0158092 10.1371/journal.pone.0158092 PMC4915721 27328124

[CR75] Wellenzohn S, Proyer RT, Ruch W (2018). Who benefits from humor-based positive psychology interventions? The moderating effects of personality traits and sense of humor. Frontiers in Psychology.

[CR77] Wilson TD, Gilbert DT (2008). Explaining away: A model of affective adaptation. Perspectives on Psychological Science.

[CR78] Xiaomei GUO, Yingchun LIU, Zhi LI, Ruibin FANG, Longdan LI, Xingjie HU, Lijuan DONG (2020). Evaluation of positive psychological interventions for people requiring temporary isolation during COVID-19 outbreak. Chinese Journal of Integrative Nursing.

