# Supplementary material for: Acting on Values: A Novel Intervention Enhancing Hedonic and Eudaimonic Well-Being
Source: J Happiness Stud. 2022 Oct 4;23(8):3889–908. doi: 10.1007/s10902-022-00585-4 (PMC9530432; doi:10.1007/s10902-022-00585-4)
Supplement: Supplementary file 1 — Supplementary file1 (DOCX 13 kb) [file 10902_2022_585_MOESM1_ESM.docx]

**The diary participants completed each week.**

We invite you to fill in the diary. Here, we will ask you to declare how often you would like to practice the meditation you received in the coming week.

Each week you will also be asked to what extent you have succeeded in implementing these plans.

Don't worry if you don't manage to realize your plans 100% - we simply want you to be honest with us.

It should not take more than 5 minutes to complete the diary each time.

1. To what extent did you manage to implement your plan from last week?

1 (0%) 2 3 4 5 6 7 8 9 10 (100%)

2. Briefly describe what you did to achieve it (what actions did you take)?

....................................................................................................................................................

2. Briefly describe what you did to achieve it (what actions did you take)?

....................................................................................................................................................

3. What value do you plan to address in the coming week?

- Self-management

- Stimulation

- Hedonism

- Achievement

- Power

- Security

- Adaptation

- Tradition

- Benevolence

- Universalism

4. Briefly describe what you intend to do to implement this value in your life (what actions do you want to take)?

Thank you for completing the diary!
