# Supplementary material for: Acting on Values: A Novel Intervention Enhancing Hedonic and Eudaimonic Well-Being
Source: J Happiness Stud. 2022 Oct 4;23(8):3889–908. doi: 10.1007/s10902-022-00585-4 (PMC9530432; doi:10.1007/s10902-022-00585-4)
Supplement: Supplementary file 2 — Supplementary file2 (DOCX 38 kb) [file 10902_2022_585_MOESM2_ESM.docx]

Supplement A

Table 1.

*Differences in well-being before and after training in experimental and control groups*

|  | *M(SD)* | | *F* | η^2^ | Post hoc |  |
| --- | --- | --- | --- | --- | --- | --- |
| Training | Pre-test | Post-test |  |  |  |  |
| Satisfaction with life | | | | | | |
| Values | 4.15(1.06) | 4.62(1.22) | 28.44^***^ | .26 | T1<T2 |  |
| Mindfulness | 4.00(1.21) | 4.72(1.21) | 87.13^***^ | .52 | T1<T2 |  |
| Control | 3.82(1.21) | 4.02(1.18) | 5.14 | .05 |  |  |
| Eudaimonic well-being | | | | | | |
| Values | 4.86(.74) | 5.06(.80) | 9.59^**^ | .11 | T1<T2 |  |
| Mindfulness | 4.91(.75) | 5.20(.76) | 35.42^***^ | .31 | T1<T2 |  |
| Control | 4.85(.79) | 4.84(.80) | .05 | .00 |  |  |
| Positive affect | | | | | | |
| Values | 3.34(.71) | 3.49(.65) | 3.29 | .04 |  |  |
| Mindfulness | 3.10(.75) | 3.53(.73) | 38.66^***^ | .33 | T1<T2 |  |
| Control | 3.07(.73) | 3.05(.78) | .03 | .00 |  |  |
| Negative affect | | | | | | |
| Values | 2.67(.91) | 2.20(.86) | 21.68^***^ | .22 | T1>T2 |  |
| Mindfulness | 2.63(.81) | 2.12(.90) | 46.73^***^ | .37 | T1>T2 |  |
| Control | 2.79(.94) | 2.82(.99) | .15 | .00 |  |  |

Note. ^*^*p* < .05, ^**^ *p* < .01, ^***^ *p* < .001; *p*‐values corrected for False Discovery Rate.

Table 3

*The interaction effects between the time and the group*

|  | Time * group interaction | | |
| --- | --- | --- | --- |
| Variable | *F* | *p* | η^2^ |
| Satisfaction with life | 9.402 | <.001 | .07 |
| Eudaimonic well-being | 9.415 | <.001 | .07 |
| Positive affect | 9.032 | <.001 | .06 |
| Negative affect | 13.157 | <.001 | .09 |

Figure 1. Satisfaction with life before and after the training in the two experimental groups and control group

Figure 2. Eudaimonic well-being before and after the training in the two experimental groups and control group

Figure 3. Positive affect before and after the training in the two experimental groups and control group

Figure 4. Negative affect before and after the training in the two experimental groups and control group
